# Rapid, Paralog-Sensitive CNV Analysis of 2457 Human Genomes Using QuicK-mer2

**DOI:** 10.3390/genes11020141

**Published:** 2020-01-29

**Authors:** Feichen Shen, Jeffrey M. Kidd

**Affiliations:** 1 Department of Human Genetics, University of Michigan, Ann Arbor, MI 48109, USA; feichens@umich.edu; 2 Department of Human Genetics and Department of Computational Medicine and Bioinformatics, University of Michigan, Ann Arbor, MI 48109, USA

**Keywords:** copy-number variation, gene duplication, k-mer

## Abstract

Gene duplication is a major mechanism for the evolution of gene novelty, and copy-number variation makes a major contribution to inter-individual genetic diversity. However, most approaches for studying copy-number variation rely upon uniquely mapping reads to a genome reference and are unable to distinguish among duplicated sequences. Specialized approaches to interrogate specific paralogs are comparatively slow and have a high degree of computational complexity, limiting their effective application to emerging population-scale data sets. We present QuicK-mer2, a self-contained, mapping-free approach that enables the rapid construction of paralog-specific copy-number maps from short-read sequence data. This approach is based on the tabulation of unique k-mer sequences from short-read data sets, and is able to analyze a 20X coverage human genome in approximately 20 min. We applied our approach to newly released sequence data from the 1000 Genomes Project, constructed paralog-specific copy-number maps from 2457 unrelated individuals, and uncovered copy-number variation of paralogous genes. We identify nine genes where none of the analyzed samples have a copy number of two, 92 genes where the majority of samples have a copy number other than two, and describe rare copy number variation effecting multiple genes at the APOBEC3 locus.

## 1. Introduction

Gene duplication is the major mechanism for the evolution of novel gene functions [1,2,3]. Copy-number and sequence variation within gene families is associated with a number of phenotypes, evolutionary adaptations, and human diseases [4]. Gene duplicates can arise through two broad mechanisms: duplication of DNA sequences and the reverse transcription and integration of RNA transcripts. For simplicity, we refer to all genes related to each other via duplication, regardless of the mechanism of origin, as paralogs. Duplications arising at the DNA level are generated via two processes: whole genome duplication events and smaller duplications restricted to segments of DNA (segmental duplications). Whole genome duplication is an ancient and continuing process that is common in plants and has played key roles in the evolution of vertebrates, jawed fishes (gnathostomes), and ray-finned fish (teleosts) [5,6]. Segmental duplication results in additional copies of a single gene or of a cluster of adjacent genes. Unequal crossing over during meiosis (nonallelic homologous recombination) can give rise to tandem duplications. Gene duplication events may juxtapose parts of genes and their associated regulatory elements, leading to novel gene sequences and functions. Over time, these processes result in the evolution of large gene families associated with a number of biological processes, including development, gene regulation, immune function, metabolism, and environmental response [4,7]. Immediately following duplication, paralog copies are identical. Unraveling the processes affecting the retention, loss, and divergence of gene paralogs remains a major focus of theoretical and empirical evolutionary biology [8]. 

In addition to the evolution of gene function, duplications impact the overall structure and function of the genome. Duplicated sequences are prone to subsequent gains and losses due to misalignment during meiosis. Variation among duplicated sequences accounts for the majority of copy-number variation found within vertebrates [9,10,11,12,13,14]. Gene conversion, or the nonreciprocal exchange of DNA that shares sequence similarity, is also an important process for the evolution of duplicated sequences. Gene conversion acts to homogenize sequences, is a mechanism that can lead to the concerted evolution of gene families [4,15], and must be accounted for in models of sequence evolution [16,17]. Gene conversion is thought to be of particular importance for the evolution of duplicated sequences on the sex chromosomes [18,19], and conversion of sequence from pseudogenes into functional genes is the molecular basis for a number of human diseases [15]. 

Many studies of gene duplicate evolution have used comparisons of genome reference assemblies to define patterns of gene gain, loss, and sequence divergence [20,21,22]. Short-read sequencing technology has enabled the rapid acquisition of genome-wide data from countless samples; however, systematic assessment of variation among gene duplicates using this data has been limited. The most commonly used signal for detecting the presence of a duplication in a sample is the increase in read depth obtained from whole genome sequencing. Such approaches have their origins in the genome-wide identification of human segmental duplications by mapping whole genome shotgun reads from the Celera genome project onto the public, clone-based human genome assembly [23]. Similar ideas have been adapted for the short sequencing reads produced by the Illumina platform, with improvements that model factors, such as local GC content, that may bias the observed sequencing depth. Typically, reads that align to multiple locations on a reference genome are randomly placed, severely complicating paralog specific analyses. Several alternative approaches based on probabilistic assignment of reads to repetitive and duplicated sequences have been developed. One method focused on detecting structural variation using discordant read pairs includes VariationHunter, which constructs consistent clusters of reads, including probabilistic assignment of reads that have multiple mappings [24]. Similarly, probabilistic approaches have been used to call ChIP-seq peaks from multiply-mapped reads [25,26]. These approaches are geared toward discovering genetic variation or functional genomics signals in repetitive sequences, and generally work by modeling the distribution of signals among multiple read placements.

A number of approaches for detecting copy-number variation using Illumina sequencing data have been developed [27]. Two existing approaches that are most relevant for the genome wide analysis of paralog variation are Genome STRiP and the tools based on the mrFAST and mrsFAST aligners. The Genome STRiP software was designed to discover and genotype copy-number variation across a population of sequenced samples [10,28]. To study multiallelic copy-number variants (CNVs) overlapping duplicated regions, Genome STRiP was extended to include reads mapping to reference locations that have identical sequence between duplication copies [28]. By considering reads mapping uniquely to a specific paralog, Genome STRiP can, in some cases, identify which paralog is duplicated or deleted. The mrFAST and mrsFAST aligners were designed to efficiently return all possible alignments on the genome for a read within a specified edit distance [29,30,31]. This enables direct assessment of the total copy-number of a DNA segment, including segments that are represented multiple times in the genome reference assembly. To supplement these mappings, defined sets of paralog-specific variants (PSVs) that are unique to specific duplication copies in the human genome reference were defined, and the depth of uniquely mapping reads at PSV positions was used to assign copy-number to specific paralogs [29]. For consistency across read sizes, and to limit the effect of masked regions on flanking coverage, this approach typically divides sequencing reads into nonoverlapping 36-bp long segments. This methodology was subsequently applied to an initial release of 159 samples from the 1000 Genomes Project [9]. This involved extracting nonoverlapping segments of 36 bp from existing reads and remapping these segments using the mrFAST or mrsFAST aligners, a computationally intensive process. Since paralog-specific counts were determined based on 36-bp read fragments with a unique mapping position that began at an annotated PSV position [9], effective paralog-specific detection was limited to samples with high coverage. This mapping-based approach was also applied to study copy-number variation in other samples, such as a set of primate genomes [12] and the collection of human genomes from the Simon’s Diversity Panel [32], revealing multiple insights into the evolution of duplicate genes in humans [33,34,35,36,37]. 

To enable the systematic assessment of gene duplication variation, we previously developed QuicK-mer, a mapping-free approach that efficiently determines copy-number in a paralog-specific manner [38]. Rather than the costly interrogation of all possible mapped read locations, QuicK-mer tabulated observed counts for pre-specified sets of sequences of length k (known as k-mers, by default we use k = 30 bp), a highly efficient approach. The use of k-mers has a long history in genomic DNA analysis, with many uses, including genome assembly [39,40], the annotation of repetitive sequences [41], the genotyping of structural variants [42], and the identification of sequences unique to a given sample [43,44]. Related mapping-free approaches have recently gained prominence in the analysis of RNA sequencing data [45,46,47], where they show superior performance and drastically increased speed [47]. 

The initial version of QuicK-mer relied on a pipeline of external tools and made use of cumbersome intermediate files. This made it difficult to construct k-mer sets for additional genome assemblies and imposed a large load on file reading and writing, which hindered the analysis of the thousands of high coverage genomes sequences that are now routinely generated. Here, we report QuicK-mer2, a stand-alone implementation that utilizes many ideas to increase efficiency, including encoding k-mers using a 2-bits-per-base scheme, indexing k-mers into memory using the DJB hash function, the use of bitwise operations to efficiently complement k-mers, and various design choices that leverage the efficiencies of 64-bit register sizes. We use QuicK-mer2 to generate paralog-specific copy number profiles based on the observed depth at informative k-mers obtained from 30X coverage sequencing of 2457 individuals produced by the 1000 genomes consortium. We describe broad-scale patterns of gene copy-number variation across humans report low-frequency copy-number variants, and present results in a publicly accessible format using the TrackHub system [48]. 

## 2. Materials and Methods 

### 2.1. Conceptual Overview of the QuicK-mer Algorithim

The QuicK-mer pipeline for paralog specific copy-number estimation has been previously described [38]. The approach has three major steps. First, a set of unique k-mer sequences are identified from a genome reference for subsequent analysis. This set can be supplemented by additional k-mers that are unique to a family of related sequences (as in [49]), or that are absent from the genome reference, including unique junction or insertion sequences (as in [50,51]). This process need only be performed once but requires many external programs. We previously released pre-built unique k-mer sets for commonly utilized genomes (https://kiddlabshare.med.umich.edu/QuicK-mer/). Second, the occurrence count for each k-mer identified in step 1 is tabulated from Illumina sequencing data generated from a sample. Third, the tabulated raw counts are normalized to correct for the effects of local GC content. The normalized counts are then converted to estimated copy number values. The results are typically summarized in bins of consecutive k-mers along the genome, with typically 1000 or 3000 k-mers per bin. These results then serve as the input for subsequent analysis. Originally, step 1 required the use of many external programs on large computer cluster, step 2 relied upon the Jellyfish [52] k-mer counter, and the whole pipeline produced many large intermediate files. 

### 2.2. Implementation of QuicK-mer2

**Figure 1:**
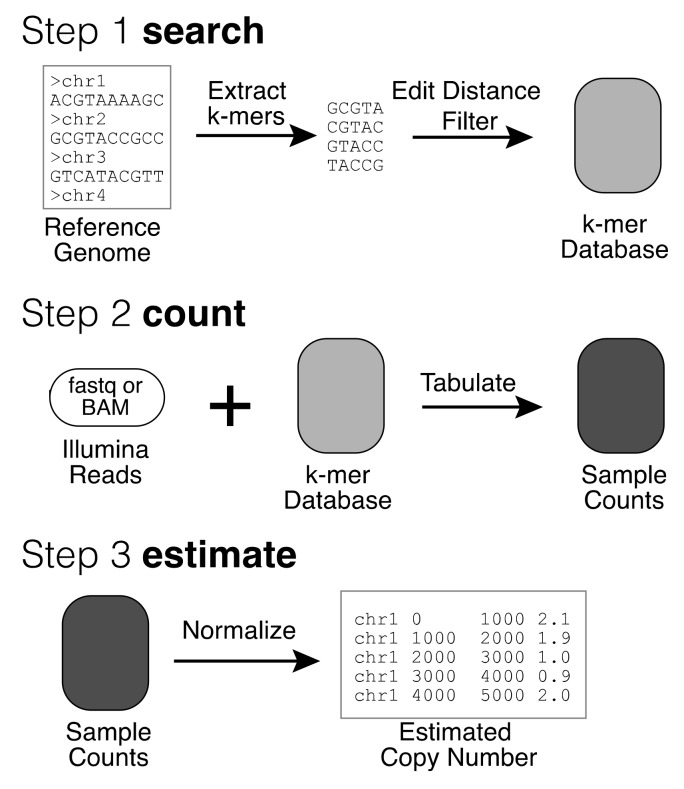
Overview of QuicK-mer2. Copy number analysis with QuicK-mer2 proceeds through three stages. First, a set of unique k-mers are identified in a reference genome. These k-mers are then further searched against the reference to find additional hits within a specified edit distance, and k-mers that have too many hits are discarded. This results in a set of k-mers for future analysis. This k-mer list is further annotated based on a user-supplied list of genomic regions unlikely to be copy number variable. K-mers in the invariable regions are used for subsequent normalization. This step is only completed once for each reference genome assembly to be analyzed (i.e., GRCh38). Second, Illumina read files from a sequenced sample are interrogated and the observed counts for each targeted k-mer are tabulated. Finally, the raw k-mer counts are normalized to account for the effects of local GC content on coverage and converted to estimated copy number values based on the specified control regions. Copy number values are output in bed format, with each interval containing an equal number of analyzed k-mers. The resulting copy number estimates are suitable for subsequent analysis.

To improve performance and simplify ease of use, we developed QuicK-mer2 as a stand-alone application that contains all three functionalities with a novel internal core (Figure 1) [53].

#### 2.2.1. K-mer Encoding and Internal Data Structures

We represent each of the four nucleotides using two bits. In the standard ASCII encoding, bits 1-2 happen to be unique among the ‘A’, ’C’, ’G’, and ‘T’ characters, with ‘A’ and ‘T’, or ‘G’ and ‘C’ differing by two. Thus, reverse complement conversion can be performed with a subtraction of the value of two in a two-bit unsigned integer space. The use of both encoding tricks and bit manipulation reduces CPU instruction cycles and avoids branching instruction execution.

With this encoding scheme, a 64-bit unsigned integer can store a maximum length of 32 nucleotides. In QuicK-mer2, the 3′-end position is encoded in the least significant bit in the integer. During stream processing, previous values can be shifted to the left two bits at a time. Any k-mer that contains the ambiguous ‘N’ character is discarded. For our applications, we consider a k-mer and its reverse complement to be equivalent. This is achieved by taking the smaller value after encoding both for hashing process. We utilize the DJB2 hash function modulo the size of the array as the hash index. An ideal hash function would generate a distinct index value for each distinct input. However, in reality, the same value can be obtained from different inputs, due to hash collision. QuicK-mer2 uses a linear probing approach, where the value colliding is appended in the adjacent array cell, to resolve hash collisions. To restrict overflow of the array, the appending direction is flipped between the upper and lower half of the array. In this collision resolving scheme, a k-mer search scan starts at the calculated hash index and moves in the indicated direction until the encoded k-mer value cell is found or stops when an empty cell is reached. Additional arrays with the same indexing strategy are also used. During k-mer enumeration from a reference genome assembly, two integer arrays are used to store the occurrence count of each k-mer and to store the number of matches during the edit distance search. Additionally, a linked list is constructed that stores the exact index of the next k-mer. This is used to rapidly reorganize the depth information into chromosomal order. 

#### 2.2.2. Edit Distance Search

To limit the effect of sequencing errors, k-mers are required to have a single exact match (the k-mer itself) as well as fewer than i matches within an edit distance of j, where j can be set to a value of 0, 1, or 2. For edit distances of j = 1 or 2, this is determined by tabulating, for each candidate k-mer, the observed occurrence in the reference of all k-mer variants that differ from the candidate k-mer by 1 or 2 substitutions. This search process is amendable to multithreading with shared memory access to the table of observed k-mer counts. By default, k-mers that are unique in the genome (a single edit-distance 0 occurrence) and have fewer than 100 near matches (edit distance 1 or 2) are retained for analysis. In addition, the search step requires a bed file listing portions of the genome unlikely to differ in copy number among the analyzed samples. This required file is provided by the user, and typically excludes nonautosomal chromosomes, known duplications, and known regions of copy-number variation. The positions indicated in the control file are used to correct observed k-mer counts based on the flanking GC content and to convert obtained counts to diploid copy-number estimates. 

#### 2.2.3. CNV Estimation

In the count step, QuicK-mer2 determines the occurrence count for each target k-mer in an input collection of sequence, typically derived from Illumina sequencing. QuicK-mer2 reads the sequence from the standard input stream, making it easy to supply inputs in FASTQ, BAM, or CRAM formats with minimal reformatting. The count step is also multithreaded, implemented as a feeder-consumer scheme where the feeder thread fetches sequencing reads and generates encoded k-mer values, while each consumer thread hashes the encoded k-mer and accumulates the depth in the corresponding index location using the lock-add CPU instruction (Figure S1).

### 2.3. Application to Data from the 1000 Genomes Project

A QuicK-mer2 k-mer list was derived for a version of the GRCh38 reference with an edit distance cutoff of j = 2. The reference version was based on the reference used by the 1000 Genomes project (ftp://ftp-trace.ncbi.nih.gov/1000genomes/ftp/technical/reference/GRCh38_reference_genome/) and included unlocalized scaffolds and decoy sequences. However, alternate loci, patch scaffolds, and HLA sequences were excluded. Regions excluded for consideration during GC correction and to set copy-number baseline included nonautosomal chromosome sequences, segmental duplications (obtained from the UCSC Genome Browser Annotation Track), and nonmobile element duplications, and deletions from [12,54,55,56,57] were obtained from the Database of Genomic Variants [58]. Regions were merged together and converted to a file of regions to include using bedtools [59]. 

CRAM format files for 30x of Illumina NovaSeq sequencing of 2504 samples from the 1000 Genomes project phase 3 sample set were generated at the New York Genome Center and downloaded from ftp://ftp.1000genomes.ebi.ac.uk/vol1/ftp/data_collections/1000G_2504_high_coverage/. Read sequences were extracted from Cram format files using samtools [60] with flags set to exclude nonprimary alignments, reads marked as duplicates, and reads that failed quality control checks (flag –F 3840). Analysis focused on a set of 2457 unrelated individuals identified by [61]. Copy-number was estimated in windows containing 1000 k-mers and converted to tracks for display using a UCSC Track Hub [48]. For visualization, heat maps were created of copy number values rounded to the nearest integer. Gene analysis was based on the hg38 UCSC Curated RefSeq set. For each gene, the longest isoform was selected and windows that intersected with each gene were identified using bedtools. This resulted in 28,013 genes that needed to be analyzed. This included 13 mitochondrial genes that were omitted from most analyses. Gene copy number estimates were based on the median value of the intersecting windows for each sample. For male samples, the copy number estimates on the nonPAR regions of the X-chromosome were doubled prior to analysis.

### 2.4. Data Availability

QuicK-mer2 can be obtained from https://github.com/KiddLab/QuicK-mer2. A track hub showing individual copy number data from all samples, as well as per-window copy-number ranges for each continental super population, is publically available at https://github.com/KiddLab/kmer_1KG. Per-gene copy number estimates are provided in the supplementary material.

## 3. Results

We developed QuicK-mer2 to enable rapid analysis of paralog specific copy-number variation from the thousands of high-coverage whole-genome sequences that had recently become available. QuicK-mer2 is a stand-alone reimplementation of our previously described and validated approach that estimates genome copy number based on counts for unique k-mers [38]. In contrast to the onerous procedure previously required, the revised approach can define a set of unique k-mers (within an edit distance of 2 substitutions) from the human genome within 256 CPU hours, a process that, with multithreading, can be completed within 12 h. Once constructed, copy number values can be estimated for this k-mer set from a ~20X human genomes in approximately 20 min using 6 CPU threads. We confirmed that k-mer counts on test data were identical to those obtained using Jellyfish [52], and verified that the comparisons with [9] described in the supplementary material of [38] were also recapitulated with the QuicK-mer2 pipeline.

To illustrate the utility of our updated approach, we constructed paralog-specific copy-number estimates using newly released 30x Illumina whole-genome sequencing of 2504 samples from the 1000 Genomes project sample set. Using a version of the GRCh38 reference that lacked alternative haplotype sequences but included decoy sequences, unplaced sequences, and the Epstein Bar Virus (EBV) genome, we identified unique 2,300,498,292 k-mers that also had fewer than 100 other occurrences in the genome within an edit distance of two substitutions. We then determined the paralog-specific copy-number in nonoverlapping windows, with each encompassing 1000 unique k-mers. K-mer counts were converted to copy-number based on the observed k-mer counts in regions that were not previously reported as copy-number variable. Since the 1000 genomes sample set contained some individuals that were close relatives, we focused our analysis on a subset of 2457 individuals that minimized these known relationships [61].

**Figure 2:**
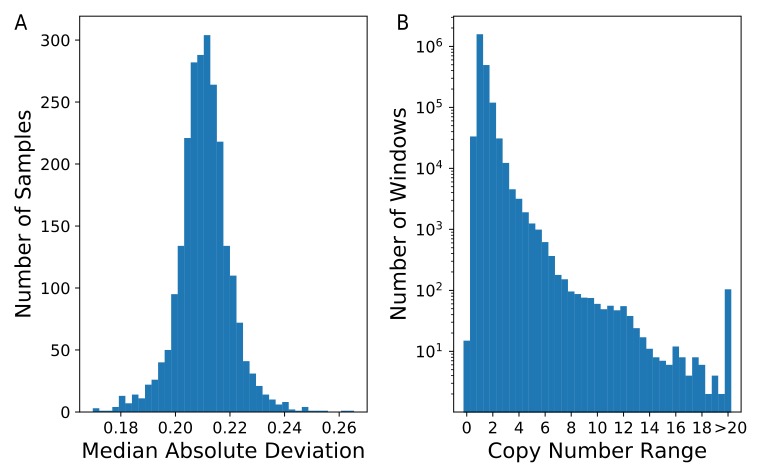
Distribution of inferred paralog-specific copy-numbers. Copy number was estimated for 2457 individuals using nonoverlapping windows that each contained 1000 k-mers. (**A**) A histogram of the median absolute deviation (MAD) calculated for all individuals is shown. (**B**) The copy-number range for each window across all samples was determined. A histogram of these ranges is shown. Note that the Y-axis is plotted on a logarithmic scale.

To assess the global variability across samples, we calculated the median absolute deviation (MAD) of the estimated copy-number in each of 2,285,696 nonoverlapping windows (Figure 2A). The distribution was centered on 0.24, with five of the 2457 individuals having a genome-wide MAD greater than 0.25. On average, 2.46% of the windows in each sample had a raw estimated copy-number less than 1.5 or greater than 2.5, with 23 samples having more than 5% of windows outside of this range. Looking across all samples, we observed that 70.7% (1,615,758 windows) of windows had a copy number range of less than 1.5, while 7.7% (176,825 windows) of windows had a copy-number range of at least 2.0 (Figure 2B). This indicates that the majority of the assayed genome had a fixed copy-number. As expected, a copy-number state of two was by far the most common across all samples, with 95.9% (2,192,153) of windows having a mean copy number estimate between 1.75 and 2.25. 

**Figure 3:**
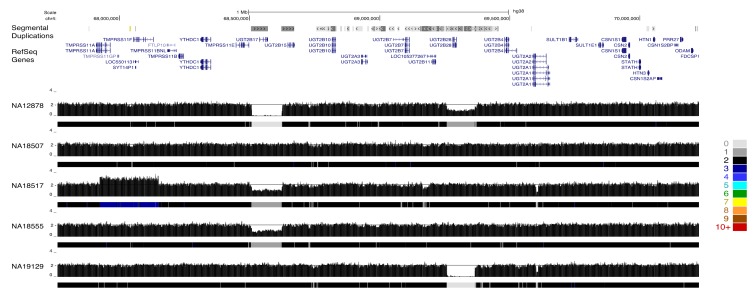
Paralog-specific CNV detection at the *UGT2B* locus. A genome browser snapshot is shown for an interval on chromosome 4 that includes several related *UGT2B* genes. Regions of segmental duplication and RefSeq gene models are indicated at the top of the image. This is followed by paralog specific copy-number profiles for five individuals. Each profile includes the raw estimated copy number in 1000 k-mer windows as well as a heatmap track depicting the estimated copy-number. The key for the heatmap track is shown to the right of the figure. Variation effecting distinct *UGT2B* paralogs is apparent. For example, sample NA12878 is estimated to contain zero copies of the *UGT2B17* gene and one copy of UGTB28, NA19129 contains two copies of *UGTB17,* and *UGT2B28* is totally absent. The authors of [9] have previously validated the variation at this locus.

The resulting paralog-specific copy-number maps represent a rich resource for identifying variation among gene duplicates. To visualize this large data set, we constructed an UCSC genome browser track hub that displayed raw copy-number estimates as well as a colored heatmap visualization. We described variation at three loci to illustrate the utility of applying QuicK-mer2 to thousands of samples. The *UGT2B* gene family plays an import role in the metabolism of xenobiotic and endogenous substances [62]. Variation among specific *UGT2B* family members on chromosome 4 has been extensively characterized using clone-based resources [9]. These results are recapitulated by QuicK-mer2 (Figure 3). Although many samples (such as NA18507) have an estimated copy number of two across the region, the specificity of QuicK-mer2 allows for the separate identification of gene content across the locus. The detected variation includes deletion of the *UGT2B17* or *UGT2B28* genes, as well as duplication of a flanking gene (*TMPRSS11F*). 

**Figure 4:**
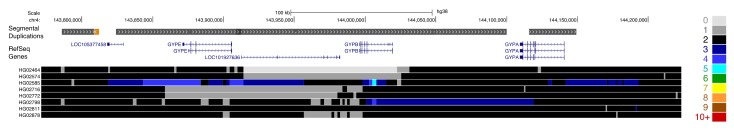
Variation at the glycophorin locus in the Gambian in Western Divisions (GWD) population. Paralog-specific copy-number estimates are shown for eight individuals at the glycophorin locus on chromosome 4. One sample, HG02811, is predicted to have a copy number of two across the entire region, matching the depiction in the genome reference assembly. The other samples show complex patterns of deletion and duplication involving portions of *GYPE*, *GYPB*, and *GYPA*. The browser display and heatmap image is formatted as depicted in Figure 3.

**Figure 5:**
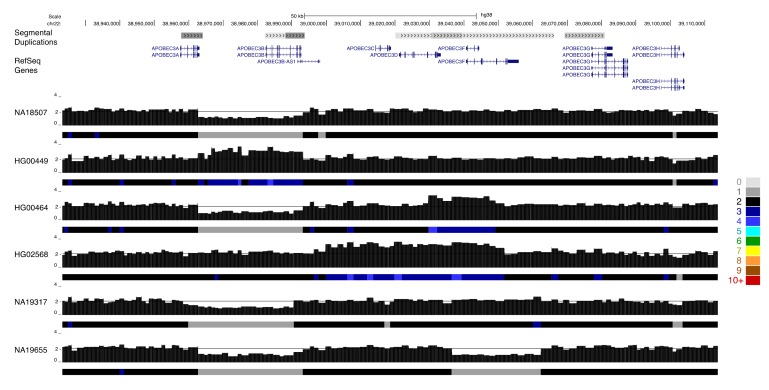
Rare copy number variation at the *APOBEC3* locus analysis of 2457 individuals identified as having experienced rare gene gain losses at the *APOBEC3* locus on chr22. Paralog specific copy number profiles are shown for six individuals. Samples NA18507 and NA19317 are heterozygous for the common *APOBEC3A/B* gene deletion polymorphism, but are predicted to carry two copies of the other *APOBEC3* genes. A duplication of the *APOBEC3A/3B* segment is present in sample HG00449. Sample HG00464 carries the common *APOBEC3A/3B* deletion as well as duplication of a portion of *APOBEC3F*, while sample NA19655 has the common *APOBEC3A/3B* deletion as well as apparent deletion of *APOBEC3F*. Individual HG02568 carries an extended duplication the includes *APOBEC3C*, *APOBEC3D*, and *APOBEC3F*.

Complex patterns of gain and loss are also apparent at medically relevant paralogs, such as the host invasion receptor genes *GYPA* and *GYPB*. Complex structural rearrangements at this locus result in gene loss and the formation of novel fusion genes, and are associated with a reduced risk of severe malaria [63]. QuicK-mer2 detects a subset of this variation in samples from the 1000 Genomes population, particularly among samples from the Gambian in Western Divisions in the Gambia collection (GWD, Figure 4). The *APOBEC3* genes on chromosome 22 are an expanded family of genes that form part of the antiretroviral innate immune system [64,65]. A deletion resulting in effective loss of *APOBEC3B* has been previously characterized and is common in some human populations [66]. Many APOBEC3 sequence variants have also been characterized. This includes *APOBEC3H* variants linked with differences in gene expression, protein stability, and HIV infectivity [67], and variation in *APOBEC3C* that leads to increased anti-retroviral activity [68]. Our survey of thousands of diverse human genomes revealed additional rare copy number variation present at the *APOBEC3* locus (Figure 5). This included an apparent reciprocal duplication of the *APOBEC3A-B* region, deletion and duplication effecting *APOBEC3F*, and a larger duplication that appeared to encompass *APOBEC3C*, *APOBEC3D*, and *APOBEC3F*.

**Figure 6:**
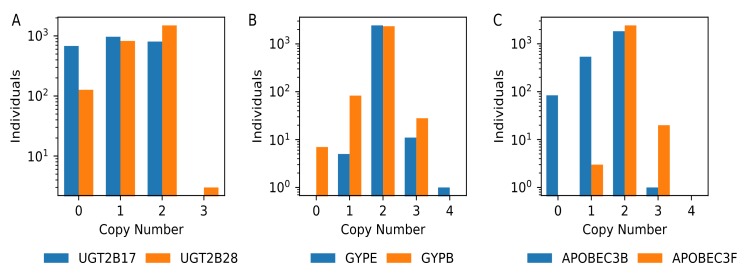
Illustrative copy number profiles for three sets of related gene paralogs. Histograms of gene copy number counts for 2457 individuals are shown for a subset of genes at the *UGT2B* locus (**A**), the *glycophorin* locus (**B**), and the *APOBEC3* cluster (**C**). Note that the Y axis is plotted on a logarithmic scale.

To systematically assess gene copy-number variation, we calculated the median value reported in windows that intersected with each gene model (Table S1) and constructed a copy number profile for each gene (Table S2, Figure 6). Omitting genes on the mitochondrial and small genes that intersected with fewer than three windows yielded 22,913 genes for comparison. Of these, we identified nine genes (*BAGE2, BMS1P14, CROCCP2, NBPF1, LINC01410, LINC01667, LOC102723780, LOC389831,* and *MGC70870*) where none of the 2457 analyzed individuals had an estimated copy number of two. This suggested that the genome reference is an inaccurate representation of the sequence content present in humans for these genes. In other cases, the genome reference assembly represents a copy number that is found in a small fraction of individuals. This includes 43 genes where at least fifty percent of the individuals have a deletion as well as 49 genes that are additionally duplicated in most individuals.

## 4. Discussion

In this manuscript, we describe the QuicK-mer2 algorithm for efficiently estimating copy number from Illumina sequencing data in a paralog-specific manner. The efficiency of this approach enables the examination of variation among gene duplicates in large collections of whole genome sequence data. To illustrate the utility of QuicK-mer2 and of the insights possible with thousands of sequenced samples, we applied our approach to newly released high coverage data from the 1000 Genomes Project to create an easily accessed resource of genome wide copy number estimates. This resource should benefit ongoing studies of gene evolution and enable the incorporation of paralog-specific variation into existing analyses. The use of the samples from the 1000 Genomes project has many benefits, including a rich set of existing genotypes of genetic variants, inclusion of samples from multiple global populations, unrestricted access to sequence data, and the availability of transformed cell lines for future studies [69]. Although our analysis shows that coverage levels of the analyzed samples are broadly similar, we cannot rule out the possible presence of artifacts associated with the transformation and cellular growth of some samples. 

QuicK-mer2 yields paralog-specific estimates of copy-number that can be used in many downstream applications. The output is suitable for analysis using a number of other existing approaches for segmenting calls into intervals that have distinct copy-number states or for clustering samples that have similar coverage levels at defined loci. The analysis in this manuscript relied upon disjoint windows of a fixed size. Although this approach is efficient and simplifies comparisons across individuals, it may be inaccurate when a variant breakpoint occurs within the boundaries of an analysis window. We attempted to limit this effect by focusing our analysis on genes that overlapped with at least three windows and by tabulating median rather than mean values. A more refined approach would utilize discrete k-mer sets associated with each gene interval rather than fixed windows. Such values can be extracted from the results produced by the QuicK-mer2 count operation. Additionally, our approach reports only genomic copy number. Further analyses are required to confirm that additional gene copies are intact and complete, and to identify rearrangement breakpoints. Applying QuicK-mer2 to linked read data produced by the 10X genomics platform may allow for the assignment of duplicate copies to specific genomic loci [70]. This may be particularly valuable for studies where the reference genome is not as complete as in humans.

Approaches for discovering structural variation from sequence data are typically dived into four categories based on the type of information they use: read pair information, read depth information, the identification of split sequencing reads, and read assembly [71]. QuicK-mer2 only uses the depth observed at predefined k-mer sequences to estimate copy number in a paralog-specific manner. The use of other signals in the data may allow for the interrogation of additional positions that are unique to duplications having high levels of sequence identity. However, such approaches would introduce an additional computational overhead.

The QuicK-mer2 method was designed for the analysis of germ line genome variation using whole genome sequencing data. With modifications, it can be applied in other contexts. The fundamental approach can be applied to the analysis of data from tumors, although the role of gene paralog variation in cancer has not been fully explored [72,73]. Although somatic sequence mutations will disrupt perfect matches at overlapping k-mers, only a small minority of the total k-mers in the genome are expected to be altered [74]. However, a new set of genomic control intervals reflecting the genomic rearrangements present in the studied cancer types would be required prior to analysis. In principle, the k-mer counting abilities of QuicK-mer2 could be applied to exome or other targeted capture data sets. However, the assumptions of the normalization process are violated in capture experiments since distinct regions are typically represented at different rates. Proper normalization therefore requires an additional locus specific depth correction. Several tools have previously implemented such approaches for detecting germline and somatic copy number variation from capture data [75,76,77].

The QuicK-mer2 approach relied upon the existence of a high-quality reference genome. Although correctly resolving duplicated sequences remains a major challenge in genome assembly, algorithmic improvements will enable the better resolution of duplicated sequences using long-read technologies [78,79]. QuicK-mer2 can also be supplemented with additional k-mers for tabulation. One application is the analysis of k-mers that are shared among a duplication family, rather than being unique to a single specific duplicate, an approach we have previously used to study amplicon sequences on the Y chromosome [49]. Custom k-mer lists can also be used to measure the presence of nonreference genomic features such as found at known structural variation breakpoints [51] or in other types of predicted insertions. 

## Supplementary Materials

The following are available online at https://www.mdpi.com/2073-4425/11/2/141/s1, Table S1: Individual level gene copy-number estimates, Table S2: Rounded gene copy-number profiles, Figure S1: Effect of multithreading on QuicK-mer2 performance.
