# Supplementary material for: Rapid, Paralog-Sensitive CNV Analysis of 2457 Human Genomes Using QuicK-mer2
Source: Genes (Basel). 2020 Jan 29;11(2):141. doi: 10.3390/genes11020141 (PMC7073954; doi:10.3390/genes11020141)
Supplement: Supplementary file 1 [file genes-11-00141-s001.zip › supp/Figure S1.pdf]

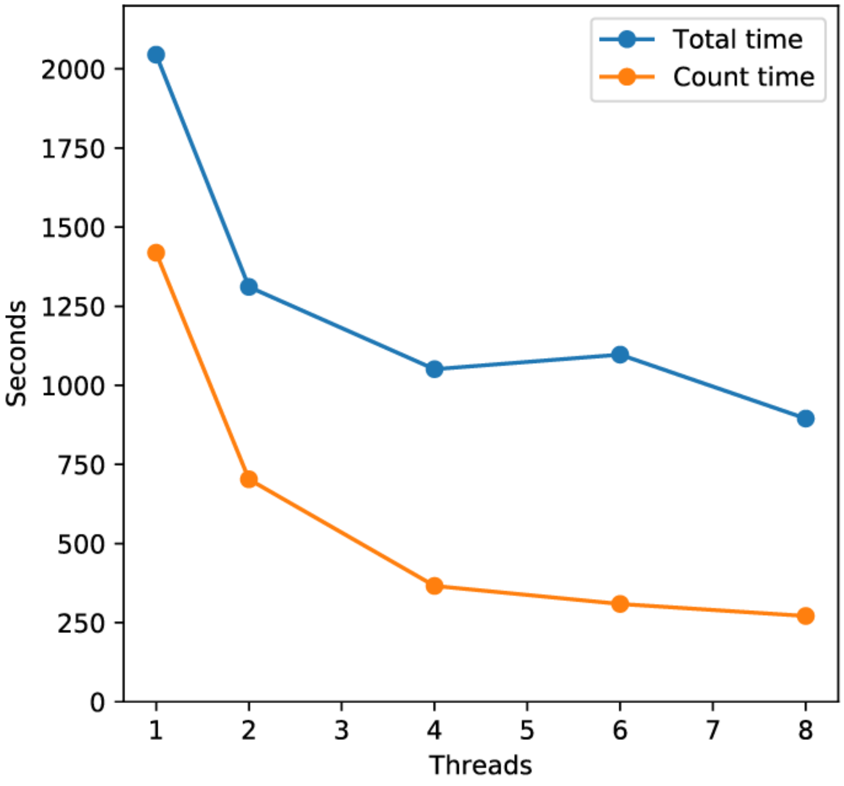

Figure S1. Effect of multithreading on QuicK-mer2 performance
Timing statistics were calculated for using one to 8 threads. The orange line shows the time used in the counting step itself, while the blue line shows the total time spent including file I/O. At around six threads the process becomes I/O bounded. The fluctuations in total time spent reflect random occurrence of disk access across the network. The plot is based on QuicK-mer2 analysis of the canine genome based on sample SRR3384067 using Xeon E7 4850 2GHz processors.
